# Optimal Extracellular Matrix Niches for Neurogenesis: Identifying Glycosaminoglycan Chain Composition in the Subventricular Neurogenic Zone

**DOI:** 10.3389/fnana.2021.764458

**Published:** 2021-10-04

**Authors:** Aurelien Kerever, Eri Arikawa-Hirasawa

**Affiliations:** ^1^ Research Institute for Diseases of Old Age, Juntendo University Graduate School of Medicine, Tokyo, Japan; ^2^ Department of Neurology, Juntendo University School of Medicine, Tokyo, Japan

**Keywords:** fractone, heparan sulfate chains, neurogenesis, subventricular zones, extracellular matrix, growth factors, glycosaminoglycans

## Abstract

In the adult mammalian brain, new neurons are generated in a restricted region called the neurogenic niche, which refers to the specific regulatory microenvironment of neural stem cells (NSCs). Among the constituents of neurogenic niches, the extracellular matrix (ECM) has emerged as a key player in NSC maintenance, proliferation, and differentiation. In particular, heparan sulfate (HS) proteoglycans are capable of regulating various growth factor signaling pathways that influence neurogenesis. In this review, we summarize our current understanding of the ECM niche in the adult subventricular zone (SVZ), with a special focus on basement membrane (BM)-like structures called fractones, and discuss how fractones, particularly their composition of glycosaminoglycans (GAGs), may influence neurogenesis.

## Introduction

**FIGURE 1:**
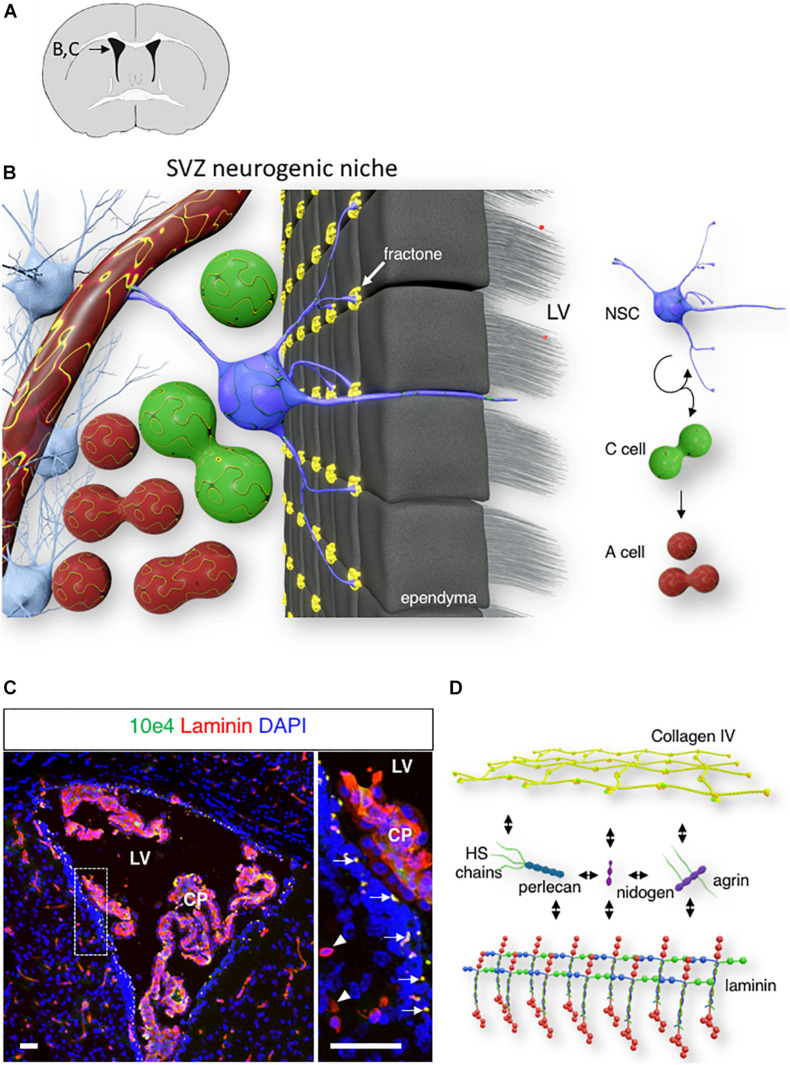
Fractone: extracellular matrix (ECM) niche in the SVZ. **(A)** A schematic of a mouse brain coronal section at bregma 0.1 mm, with an inset displaying the localization of the lateral ventricle shown in panels **(B,C)**. **(B)** 3D rendering of the SVZ neurogenic niche showing a single NSC (blue) contacting the ventricle lumen, a capillary, and numerous fractones (yellow). **(C)** Confocal image of the lateral ventricle displaying laminin (red) and N-Sulfated HS epitope 10E4 (green) immunoreactivity in the SVZ. Arrows indicate the fractones that are immunoreactive for both laminin and N-sulfated HS epitope 10E4. Arrowheads indicate laminin-immunoreactive SVZ capillaries. Scale bar: 50 μm. **(D)** A schematic of major fractones ECM component.

In the adult mouse brain, neurogenesis occurs continuously in the subventricular zone (SVZ) of the lateral ventricle (Altman, 1963; Doetsch et al., 1997) and the subgranular zone of the hippocampal dentate gyrus (Seki and Arai, 1993; Eriksson et al., 1998). In the adult SVZ, type B stem cells give rise to type C transit-amplifying cells, which, in turn, produce type A neuroblasts (Doetsch, 2003). These neuroblasts migrate toward the olfactory bulb along the rostral migratory stream, where they mature into GABAergic interneurons (Lois and Alvarez-Buylla, 1994; Alvarez-Buylla et al., 2002; Kriegstein and Alvarez-Buylla, 2009). The complex microenvironment that supports this series of events is commonly referred to as the neurogenic niche. This niche consists of various cell types that surround neural stem cells (NSCs), such as neural stem and progenitor cells, ependymocytes, mature and immature neurons, and astrocytes, as well as the vasculature. The extracellular matrix (ECM) is another critical component of this niche. Notably, NSCs have been shown to contact the basement membrane (BM) of the vasculature at sites lacking astrocyte endfeet and pericyte coverage (Tavazoie et al., 2008). In addition, vascular BM NSCs also contact a local ECM structure called fractones (Figures 1A,B).

## Fractone: Extracellular Matrix Niche in the Subventricular Zone

Fractones are extravascular ECM structures that are localized along the ventricular wall. These structures were initially observed through laminin immunostaining as small punctate structures of 2–5 μm, located behind the ependyma (arrow, Figures 1A–C). However, transmission electron microscopy revealed that fractones are electron-dense structures with branched morphology that allow them to contact numerous surrounding cells, including ependymocytes, astrocytes, NSC, and progenitor cells (Mercier et al., 2002, 2003). The ependymal wall contains interstitial clefts that allow the diffusion of signaling molecules from the cerebrospinal fluid (Brightman, 2002). Fractones are located at the end of these narrow channels and are ideally placed to receive growth factors and cytokines produced by the choroid plexus (Kerever et al., 2007; Mercier, 2016). Fractones first appear around postnatal day 5 and are composed of a ubiquitous BM component (Kerever et al., 2007; Nascimento et al., 2018; Sato et al., 2019). The presence of fractones rich in BM protein may participate in increasing the tissue stiffness of the neurogenic niche (Kjell et al., 2020). Recent studies have proposed ependymocytes (Nascimento et al., 2018) and GFAP-expressing cells (Sato et al., 2019) as cells that produce fractones. This suggests that the formation of fractones results from the contribution of various cells in the niche.

While fractone protein composition closely resembles that of the vascular BM, the fractone heparan sulfate (HS) composition is unique. HS chains belong to the Glycosaminoglycans (GAGs) family. GAGs are long, unbranched, hydrophilic, highly charged chains composed of repeating disaccharide units that can be classified into four groups based on their core disaccharide structure: keratan, hyaluronan, chondroitin sulfate/dermatan sulfate, and HS. Only HS chains can be found in vascular BM and fractones. N-sulfated HS chains recognized by 10E4 epitope immunoreactivity suggests that fractones HS present higher levels of sulfation than HS from the vascular BM (Figure 1C; Kerever et al., 2007).

Fractones are composed of ubiquitous BM components. Collagen type IV, the most abundant component of the BM, forms a network-like structure and is linked to a network of laminins with the help of nidogen/entactin (Pozzi et al., 2017). In addition, fractones contain two major types of heparan sulfate proteoglycans (HSPGs), perlecan and agrin (Figure 1D). The other main member of the BM type of HSPG, collagen XVIII, remains undetected in either fractones or vascular BM in the SVZ (Kerever et al., 2007).

Fractones may play various roles in the neurogenic niche through laminin-integrin interactions (Shen et al., 2008; Nascimento et al., 2018; Sato et al., 2019) by regulating heparin-binding ligand availability (Kerever et al., 2007) in the niche and promoting growth factor signaling (Douet et al., 2012; Kerever et al., 2014; Mercier and Douet, 2014). NSC interaction with laminin through α 6 β 1 integrin expressed on its cell surface is essential to maintain NSC quiescence (Shen et al., 2008). Laminins are heterotrimeric glycoproteins composed of 1 α, 1 β, and 1 γ chains. The β and γ chains coil around the α chain to form a cross-like structure with three short and one long arms. Short arms are responsible for self-polymerization and interactions with other BM molecules (Hohenester and Yurchenco, 2013). The longer arm most notably interacts with integrin and dystroglycan on the cell surface, leading to cytoskeleton rearrangement and impacting cell behavior. Various laminin isoforms can be found in fractones and the vascular BM in the neurogenic niche. While laminin α1 and γ2 are absent from both fractones and the vascular BM, laminin α5, β1/2, and γ1 are present in both. In addition, laminin α2 and α4 are present only in the vascular BM, but α3 is only present in fractones (Kerever et al., 2007; Nascimento et al., 2018; Sato et al., 2019). Other glycoproteins such as secreted modular calcium-binding protein 1 and 2 (SMOC1/2) and the laminin-related molecule netrin 4, which shares homology with the N-terminal portion of laminin β1, (Sun et al., 2011) have also been detected in fractones (Sato et al., 2019).

The capacity of fractones to specifically capture heparin-binding growth factors from the extracellular milieu highlights a critical role for its HSPG perlecan and agrin. Perlecan, also referred to as HSPG 2, is a major BM type of HSPG, and deficiency of perlecan causes perinatal lethality in mice and humans (Arikawa-Hirasawa et al., 1999, 2001). The core protein is composed of five distinct domains and interacts with a variety of molecules from the ECM (laminin, nidogen, collagen IV; Figure 1D). Through its protein core and its HS chains, perlecan is involved in numerous biological processes, including embryonic development, tissue homeostasis, and pathology (Gubbiotti et al., 2017; Yu et al., 2017). In the neurogenic niche, perlecan is present in both vascular BM and fractones, and we previously reported that the presence of perlecan in fractones through its HS chains promoted FGF-2 stimulation of neurogenesis (Kerever et al., 2014).

Agrin is another major HSPG component of the BM, and plays a critical role in the hematopoietic stem cell niche (Mazzon et al., 2011; Pozzi et al., 2017). Agrin is also a key component of the microenvironment that regulates synapse differentiation at the neuromuscular junction (Gautam et al., 1996) and in neurons of the hippocampus (Böse et al., 2000), as well as in newborn neurons of the olfactory bulb (Burk et al., 2012). In the SVZ, agrin is found in both the vascular BM and fractones (Kerever et al., 2014).

In addition to the cell/ECM interaction that plays a role in maintaining NSC quiescence through laminin/integrin signaling, fractones also play a role in regulating growth factor signaling. Both perlecan and agrin bear HS chains, and might therefore contribute to the regulation of heparin-binding growth factor signaling in the SVZ.

## Heparan Sulfate Chain Structures Regulate Growth Factor Signaling

**FIGURE 2:**
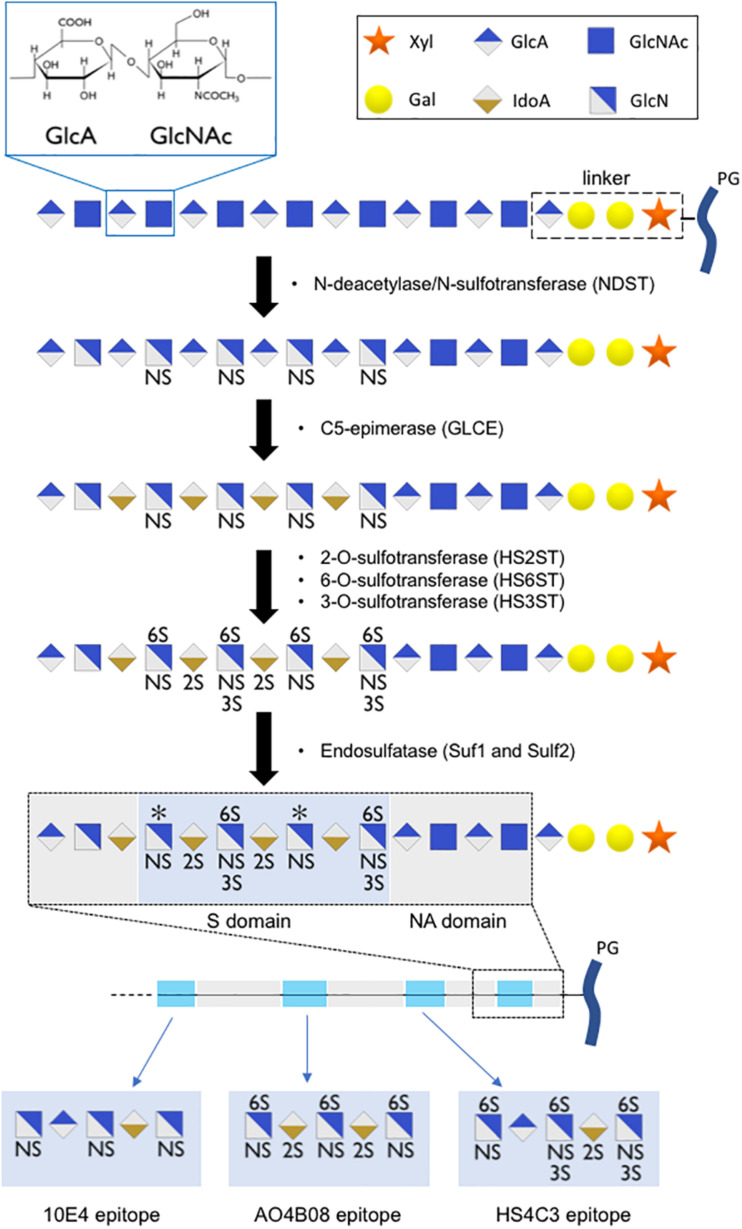
Scheme of HS chain biosynthesis. After sequential addition of glucuronic acid (GlcA) residue and an N-acetylglucosamine (GlcNAc) residue onto a linker composed of one xylose residue (Xyl), two galactose residues (Gal), and one glucuronic acid residue, the polysaccharide undergoes a series of modifications (N-sulfation, epimerization, 2O-, 6O-, 3O-sulfation) in the Golgi apparatus, leading to the formation of highly sulfated domains with precise sulfation patterns. An additional level of HS modification occurs in the extracellular space, where secreted endosulfatases can catalyze the specific 6O-desulfation of HS chains, leading to the formation of long polysaccharide chains with high sulfation heterogeneity. We proposed short oligosaccharide sequences recognized by 10E4, AO4B08, and HS4C3 epitopes.

The building blocks of HS are glucuronic acid (GlcA) and N-acetylglucosamine (GlcNAc). They are alternatively transferred to a linker composed of one xylose residue, two galactose residues, and one GlcA residue. This polysaccharide subsequently undergoes extensive modification in the Golgi apparatus, which is catalyzed by a series of enzymes. First, N-deacetylase/N-sulfotransferase (NDST) acts on a subset of GlcNAc residues to produce N-sulfated glucosamine (GlcNS). This enzyme also generates a small number of N-unsubstituted glucosamine residues due to incomplete N-sulfation. Then, a glucuronyl C5-epimerase (GLCE) acts on the GlcA residue to create Iduronic acid (IdoA), followed by the action of HS 2-sulfotransferases that catalyze the transfer of a sulfate to the C2-position of uronic acid residues. Subsequently, HS 6-sulfotransferases (HS6ST) catalyze the transfer of sulfate onto the C6 position of the glucosamine residue in HS. Finally, HS 3-sulfotransferases (HS3ST) can transfer sulfate to the 3-OH position of the glucosamine residues of HS (Sugahara and Kitagawa, 2002; Kreuger and Kjellén, 2012). Upon release into the extracellular space, secreted endosulfatase (Sulf1 and Sulf2) can catalyze the removal of a subset of 6O sulfated group from the HS chains. The modification reactions in heparan sulfate biosynthesis occur in clusters along the polysaccharide, resulting in a highly sulfated region (S domain) separated by regions devoid of sulfate (NA domain) (Figure 2). Together, these steps contribute to the formation of a sulfated polysaccharide with tremendous chemical heterogeneity, allowing HS chains to specifically interact with a wide range of molecules (Annaval et al., 2020).

Binding of HS to a ligand may impact signaling in various ways, thereby key stem cell function (Ravikumar et al., 2020). In addition, extracellular modification of HS sulfation by endosulfatase can actively modulate growth factor signaling.

### 6O-Sulfation

6O-sulfation is regulated both during biosynthesis by HS6ST enzymes that add a sulfate group to the glucosamine residue and post synthetically in the extracellular space by endosulfatase that can remove a subset of 6O sulfate group (Annaval et al., 2020). Level of 6O-sulfation than has a great impact on numerous cell-signaling pathways.

For instance, 6O-sulfated HS bind Wnt with high affinity and thereby negatively regulate Wnt activity by preventing access to its receptor (Frizzled) on the cell surface. Removal of 6O-sulfation has been shown to reduce Wnt affinity for HS, allowing the formation of an HS/Wnt/Fz complex (Ai et al., 2003). Such regulation has great implications in the neurogenic niche, as Wnt has been shown to play a critical role in regulating the fate of NSCs (Hirota et al., 2016; Kriska et al., 2016).

A similar mechanism has been reported for bone morphogenetic protein (BMP). The BMP antagonist Noggin binds to highly sulfated HS, but Sulf activity leads to the release of Noggin and restores BMP signaling (Viviano et al., 2004). Noggin expressed by ependymocytes has been shown to promotes neurogenesis by blocking BMP signaling (Lim et al., 2000). BMP4 and BMP7 have been shown to specifically bind to Fractones HS and inhibit cell proliferation in the neurogenic niche (Douet et al., 2012; Mercier and Douet, 2014).

Another strategy involves HS acting as a coreceptor with FGF-2 as a prime example. HS is necessary for the formation of the ternary complex of basic fibroblast growth factor (FGF-2), FGF receptor (FGFR), and HS (Mohammadi et al., 2005).

6O-sulfation is not required for FGF2 binding to HS, but it is necessary for the formation of the ternary complex and subsequent cell signaling (Guimond et al., 1993; Pye et al., 1998). Therefore, Sulf provides the possibility to finely tune FGF-2 activity by converting a coreceptor type of HS into one that stores FGF-2 and prevents downstream signaling. In the neurogenic niche, FGF-2 specifically binds to fractones HS, and this interaction is necessary for FGF-2 stimulation of cell proliferation (Kerever et al., 2007; Douet et al., 2013).

Regulation of HS capacity to act as a co-receptor by Sulf has also been reported for numerous other growth factors, including amphiregulin (Narita et al., 2007), hepatocyte growth factor (HGF; Lai et al., 2004), heparin-binding epidermal growth factor-like growth factor (HB-EGF; Lai et al., 2003), FGF-1, stromal cell-derived factor-1 (SDF-1), and vascular endothelial growth factor (VEGF; Uchimura et al., 2006). These results demonstrate that the regulation of 6O-sulfation alone can impact numerous signaling pathways. 6O-sulfation was detected using the anti-HS phage display antibody AO4B08. This antibody recognizes a short oligosaccharide sequence that includes N-sulfation, C5-epimerization, 2O-sulfation, and high levels of 6O-sulfation (Dennissen et al., 2002; ten Dam et al., 2003; Kurup et al., 2007). A short oligosaccharide sequence recognized by AO4B08 antibody is presented in Figure 2. We recently reported that fractones display high AO4B08 immunoreactivity (Kerever et al., 2021). Vascular BM in the SVZ displayed weaker AO4B08 immunoreactivity. In addition, it is noteworthy that AO4B08 immunoreactivity in fractones is heterogeneous. Thus, fractones on the dorso-lateral side of the ventricle displayed stronger AO4B08 immunoreactivity than fractones on the ventral side of the ventricle (Kerever et al., 2021).

### 3O-Sulfation

HS 3-sulfotransferases enzymes that catalyze the transfer of a sulfate on the 3O-position of the glucosamine residue have been shown to display different subcellular localizations. In particular, HS3ST2 can be found in the plasma membrane, while HS3ST3B resides in the Golgi apparatus (Delos et al., 2018). This difference in localization may result in the formation of distinct sulfation motifs. 3O-sulfation has been reported to modulate ligand binding (Chopra et al., 2021). Detection of 3O-sulfation can be performed using the anti-HS phage display antibody HS4C3 (van Kuppevelt et al., 1998; ten Dam et al., 2006; Hirano et al., 2012). HS4C3 recognizes a short oligosaccharide sequence that includes N-sulfation, C5-epimerization, 2O-sulfation, 6O-sulfation, and 3O-sulfation. A short oligosaccharide sequence recognized by HS4C3 antibody is presented in Figure 2. We recently reported that fractones displayed strong HS4C3 immunoreactivity (Kerever et al., 2021).

## Aging of the Neurogenic Niche

Fractones have been shown to be altered under some pathological conditions. In the SVZ of BTBR t + tf/J mice, a mouse model of autism spectrum disorder, fractones were reported to be drastically reduced in size and number (Mercier et al., 2011, 2012). In contrast, the size of fractones has been reported to increase following long-term hydrocephalus in adult mice (Campos-Ordoñez et al., 2014). In addition, we previously reported that the structure and composition of fractones were altered in aged mouse SVZ (Kerever et al., 2015; Yamada et al., 2017). With aging, neurogenesis declines, (Maslov et al., 2004) and the neurogenic niche undergoes structural and functional remodeling (Luo et al., 2006; Rojas-Vázquez et al., 2021). Ependymal cells present altered morphology, their number decreases while the number of astrocytes interposed between ependymocytes increases (Luo et al., 2008; Capilla-Gonzalez et al., 2014). Additionally, the blood brain barrier in SVZ capillaries is compromised, leading circulating pro-inflammatory molecules to potentially affect the niche (Obermeier et al., 2013; Segarra et al., 2021).

Fractone size gradually increases with age (Kerever et al., 2015; Nascimento et al., 2018). In addition, the HS composition of fractones was also modified in the aged SVZ. We previously reported on disaccharide analysis of the young and aged SVZ that total 6O-sulfation decreased in the aged SVZ. This loss of 6O-sulfation was accompanied by impaired FGF-2 signaling (Yamada et al., 2017). The aged fractones also displayed reduced immunoreactivity for the N-sulfated epitope 10E4 (Kerever et al., 2015) and reduced AO4B08 immunoreactivity (Kerever et al., 2021). This suggests dramatic changes in the sulfation of fractones HS, and these changes may affect growth activity and participate in the age-related decline of neurogenesis.

## Conclusion

As we have described, minute modification of sulfation along the HS chains leads to dramatic changes in HS regulation of growth factor signaling. Therefore, it is critical to identify strategies to reveal precise HS sequences. The biochemical approach, which consists of breaking apart the HS chains into disaccharide units, is helpful to obtain broad information pertaining to HS composition, but it fails to elucidate the actual organization within the HS chain and cannot provide information on HS heterogeneity in the microenvironment. As we previously reported in the context of the SVZ, immunoreactivity for 10E4 shows that HS displays dramatically different sulfation signatures in the vascular BM than in fractones. The development of specific anti-HS antibodies is a great tool for deciphering the HS code (van Kuppevelt et al., 1998; Dennissen et al., 2002; Thompson et al., 2009), and studying the influence of local changes in HS composition on cell signaling in health and pathological conditions.

## Author Contributions

AK and EA-H conceptualized the manuscript. Both authors drafted the manuscript and have reviewed and agreed with the publication of the manuscript.

## Conflict of Interest

The authors declare that the research was conducted in the absence of any commercial or financial relationships that could be construed as a potential conflict of interest.

## Publisher’s Note

All claims expressed in this article are solely those of the authors and do not necessarily represent those of their affiliated organizations, or those of the publisher, the editors and the reviewers. Any product that may be evaluated in this article, or claim that may be made by its manufacturer, is not guaranteed or endorsed by the publisher.
